# Use of the QuantiFERON Monitor Assay to Predict Clinical Outcomes in Solid Organ and Hematopoietic Cell Transplant Recipients: A Scoping Review

**DOI:** 10.1111/tid.70074

**Published:** 2025-07-01

**Authors:** Bradley J. Gardiner, Roy F. Chemaly, Vladyslav Nikolayevskyy, Riccardo Alagna, Davide Manissero, Camille N. Kotton

**Affiliations:** ^1^ Department of Infectious Diseases Alfred Health and School of Translational Medicine Monash University Melbourne Australia; ^2^ Department of Infectious Diseases, Infection Control and Employee Health Division of Internal Medicine The University of Texas MD Anderson Cancer Center Houston Texas USA; ^3^ QIAGEN Manchester Ltd Manchester UK; ^4^ QIAGEN S.r.l. Milan Italy; ^5^ Transplant and Immunocompromised Host Infectious Diseases Infectious Diseases Division Massachusetts General Hospital Harvard Medical School Boston Massachusetts USA

**Keywords:** immunosuppression, infection, QuantiFERON Monitor, solid organ transplant

## Abstract

**Background:**

QuantiFERON Monitor (QFM) is an interferon‐gamma release assay designed to provide a global measure of innate and adaptive cell‐mediated immune function. We performed a scoping review to explore published evidence on the ability of QFM to predict clinical outcomes in solid organ transplant (SOT) and allogeneic hematopoietic cell transplant (HCT) recipients.

**Methods:**

A literature search was conducted using Embase, Cochrane, and PubMed databases and included studies reporting QFM values and the incidence of infection and/or organ rejection or graft‐versus‐host disease (GvHD).

**Results:**

Thirteen publications (9 SOT, 4 HCT) were included. Among SOT studies, infection (*n* = 8), rejection (*n* = 3), mortality (*n* = 2), and immunosuppression regimens (*n* = 7) were assessed as outcomes; low QFM values were associated with increased infection risk in 7/8 studies. Correlations were also identified between immunosuppression regimens and QFM results in 6/7 studies. No clear relationships between QFM values and rejection or mortality could be determined, possibly due to the low number of events reported. Infection (*n* = 4), GvHD (*n* = 3), and mortality (*n* = 1) were assessed in the HCT studies, with 3/4 reporting an association between low QFM values and infection. There was a high degree of heterogeneity in the transplant populations, outcome measures and definitions, QFM thresholds, and timing of sample collection.

**Conclusions:**

The available data suggest that QFM may be able to identify overly immunosuppressed individuals at higher risk of infection; additional studies are needed to further evaluate its predictive utility in transplant and other settings.

## Graphical abstract

QuantiFERON‐Monitor (QFM) is an interferon‐gamma release assay that could measure the level of immunosuppression and predict infections in transplant recipients. We reviewed 13 published studies (9 solid organ, 4 hematopoietic cell recipients). Low QFM values were associated with infection in 7/8 studies, and correlations with immunosuppression were reported in 6/7.

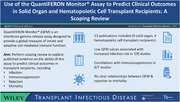

**Abbreviations**
AUROC: area under the receiver operating characteristic curve
CI: confidence interval
CMV: cytomegalovirus
EBV: Epstein–Barr virus
GvHD: graft‐versus‐host disease
HCT: hematopoietic cell transplant
HR: hazard ratio
IFN‐γ: interferon‐gamma
OR: odds ratio
QFM: QuantiFERON Monitor
SOT: solid organ transplant

## 1 Introduction

Solid organ transplant (SOT) and allogeneic hematopoietic cell transplant (HCT) are life‐saving interventions for many serious diseases [1, 2]. Survival following transplant is dependent on long‐term immunosuppression. The level of immunosuppression must be intricately balanced; however, since insufficient immunosuppression may cause organ rejection or graft‐versus‐host disease (GvHD), while excessive immunosuppression can result in infections and malignancy [2, 3, 4, 5]. Thus, monitoring and optimizing the level of immunosuppression is critical.

The net state of immunosuppression is subject to interindividual variability which is influenced by a number of factors, including age, immunosuppressive therapies, concomitant infections, and comorbidities [2, 6, 7, 8]. The current standard of care involves administration of immunosuppressive drugs at empirically defined doses, guided by time since transplant, calcineurin or mammalian target of rapamycin inhibitor levels, and history of infection, rejection, and/or GvHD [6, 9, 10, 11]. Currently, there are no standardized methods for quantifying the net state of immunosuppression in SOT and HCT recipients [7, 12, 13]. Novel pathogen‐agnostic “global” immune biomarkers offer the potential to measure the level of immunosuppression posttransplant and predict individual clinical outcomes before they occur, allowing for tailoring of immunosuppressive treatment and other preventative measures [6, 14, 15, 16, 17]. A number of assays are now available and have been explored, particularly in SOT recipients [7, 14, 18]. Thus far, their clinical use has been limited due to challenges in interpreting results and applying them at the individual patient level. As a result, there remains an unmet need for a functional assay that incorporates both innate and adaptive immune components to measure the net state of immunosuppression and inform clinical decision‐making [7, 13, 19].

QuantiFERON Monitor (QFM; QIAGEN, Hilden, Germany) is an in vitro diagnostic assay that measures interferon‐gamma (IFN‐γ) release in plasma following stimulation of whole blood samples with polyclonal immune response stimulants (anti‐CD3, a T‐cell stimulant and R848, a Toll‐like receptor 7/8 ligand) [20]. It was designed to provide a global measure of overall innate and adaptive cell‐mediated immune function in immunocompromised populations. The QFM package insert recommends the following IFN‐γ thresholds to divide patients into categories of immune function: low, < 15 IU/mL; medium, 15–1000 IU/mL; and high, > 1000 IU/mL [20]. A growing number of clinical studies have evaluated the ability of the QFM test to measure the global level of immunosuppression and predict clinical outcomes in SOT and HCT recipients [6, 13, 14, 19, 21, 22, 23, 24, 25, 26]. The overall suitability of the QFM test for these tasks, however, has yet to be fully elucidated.

In this scoping review, we have collated and evaluated the published evidence on the ability of the QFM test to predict clinical outcomes in SOT and HCT recipients. We have also explored the use and reporting of clinical outcome measures in these populations.

## 2 Methods

This scoping review was conducted in accordance with the methodological framework proposed by Arksey and O'Malley (2005) [27] and the Joanna Briggs Institute methodology for scoping reviews [28, 29]. Results are reported in alignment with the Preferred Reporting Items for Systematic Reviews and Meta‐Analysis extension for Scoping Reviews [30]. A protocol was developed to outline the methods and define the scope of the review.

### 2.1 Eligibility Criteria

Publications were included if they met the following criteria: (1) reporting of original data on the use of the QFM test; (2) a study population consisting of at least 5 SOT and/or HCT patients; and (3) reporting data on the association between QFM results and the incidence of infection and/or organ rejection or GvHD within the first year posttransplant. We excluded studies that were not published in English, did not report on associations between QFM and clinical outcomes, did not include SOT and/or HCT patients, and those reporting nonoriginal data, such as reviews, editorials, and opinion pieces. We also excluded congress abstracts. There was no limitation placed on publication date.

### 2.2 Search Strategy

The search strategy was developed in conjunction with an information specialist and involved both free‐text terms (including synonyms, abbreviations, variant spellings, etc.) and indexing terms (i.e., Medical Subject Headings). Boolean operators were used to create the final search query. Search terms included the following phrases and related terms: “QuantiFERON Monitor,” “QuantiFERON‐Monitor,” “QF monitor,” and “QF‐monitor.” Database searches were initially conducted in September 2023 of MEDLINE (PubMed), Embase, and Cochrane CENTRAL, with updated searches performed in July 2024 and March 2025. The complete search strategies for each database can be found in Table S1.

A second search using identified keywords was conducted across the following clinical trial registries: Current Controlled Trials in the metaRegister of Controlled Trials (https://www.isrctn.com/); ClinicalTrials.gov from the US National Institutes of Health database (http://clinicaltrials.gov); and the WHO Network of Collaborating Clinical Trial Registers (https://trialsearch.who.int/). Finally, the reference lists of relevant publications identified during the review were searched to capture additional relevant studies.

Following the search, the list of identified publications was collated within EndNote (Clarivate Analytics, Pennsylvania, USA), duplicates were removed, and the remaining publications were uploaded into Covidence (Veritas Health Innovation Ltd, Victoria, Australia), a web‐based platform for review management, for screening.

### 2.3 Screening

The titles and abstracts of each publication were screened by two research assistants (independent of the author team) using the pre‐established inclusion and exclusion criteria. Publications that were considered suitable for inclusion were retrieved in full and linked to their relevant citation within Covidence. The full text of each publication was assessed in detail, also by two independent reviewers. Reasons for exclusion at full‐text screening were recorded and reported in the scoping review flowchart. Conflicts and disagreements were resolved by consultation between the two reviewers and a third member of the independent review team, if needed.

### 2.4 Data Extraction

Relevant data were identified and extracted into a bespoke form, developed in Excel, by two non‐author research assistants. The form was initially tested on four publications to determine feasibility and highlight any issues with the search criteria. The form was then amended accordingly and data were extracted from all publications. Both reviewers then assessed the captured findings to ensure agreement in the data extracted, with any conflicts or disagreements resolved by a third member of the review team. The extracted data were organized in tabular form in a manner that aligned with the aim and questions of the scoping review. Key results of individual studies were summarized, with results tables reporting on the associations between QFM results and clinical outcomes including infection, rejection, and mortality. The relationships between immunosuppression regimens, including different types and doses of immunosuppressive therapy, and QFM results were also included.

## 3 Results

### 3.1 Search Results

**FIGURE 1:**
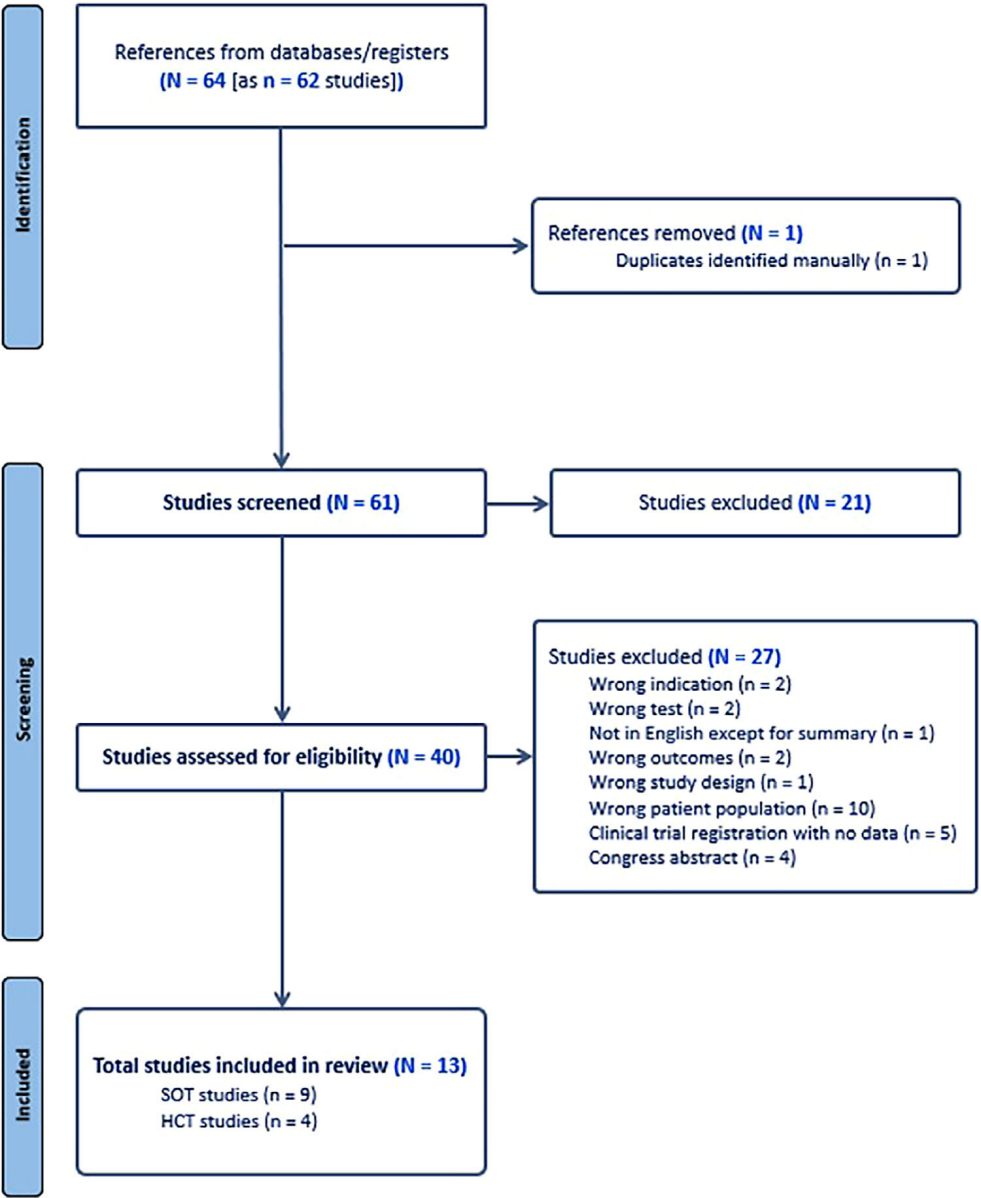
Flow diagram of search results. HCT, hematopoietic cell transplant; SOT, solid organ transplant.

The literature search identified 64 publications of potential relevance (Figure 1). After duplicates were removed, 61 unique studies underwent title and abstract review. Of these, 40 publications were reviewed in full, 27 of which were excluded from the scoping review. The 13 included publications were original manuscripts. Nine publications included information on SOT recipients and 4 on HCT recipients. The publications included information on 829 patients in total, with the majority being SOT recipients (*n* = 649 [78%]; HCT, *n* = 180 [22%]).

### 3.2 SOT Recipients

#### 3.2.1 Characteristics of Included Studies

The 9 studies included in the review comprised several different SOT populations, including 277 kidney, 226 liver, 145 lung, and 1 small bowel recipients (Table 1). The number of recipients in each study ranged from 15 to 137. In all studies, samples were collected posttransplant with 5 studies also collecting samples prior to transplant [6, 14, 21, 23, 26]. The timing of sample collection was variable and included either timeframes or specific timepoints within a range of follow‐up periods (Table 1). Five studies measured changes in clinical outcomes by collecting samples at multiple timepoints or timeframes [6, 14, 23, 24, 31]. Four studies either did not provide details on the specific timing of sample collection or were conducted as cross‐sectional studies [19, 21, 26, 32].

**TABLE 1:** Overall findings of the 9 identified studies that evaluated QuantiFERON Monitor in solid organ transplant recipients.

							Associations with clinical outcomes				
Study	Study design	Objectives	Population	Timing of sampling	Timepoints/timeframes assessed	Follow‐up duration posttransplant	Immunosuppression	Infection	Rejection	Mortality	Study conclusions
Fernandez‐Ruiz et al. [31]	Prospective observational cohort	Assess functional immune status using QFM and correlate with infection and cancer outcomes	126 kidney	Post‐transplant	Weeks 2, 4, 12, 16, 24 post‐transplant	1 year	Yes	Partial	Not reported	Not reported	QFM may have a role in the prediction of bacterial and opportunistic infection in kidney transplant recipients early post‐transplant
Gardiner et al. [14]	Prospective observational cohort	Evaluate QFM as a biomarker of immunosuppression and predictor of infection and rejection	80 lung	Pre‐ and post‐transplant	2–6 weeks, 6 weeks–3 months, 3–6 months, and 6–12 months post‐transplant	1 year	Yes	Yes	No	Not reported	QFM could be used to identify overly immunosuppressed patients post‐transplant who could be targeted for dose reduction
Margeta et al. [19]	Cross‐sectional	Compare QFM results in stable kidney transplant recipients and those with infection	71 kidney	Post‐transplant	—	Mean follow‐up 2.7 years	Yes	Yes	Not reported	Not reported	The QFM test could be useful to help guide immunosuppression dosing in kidney transplant recipients
Marx et al. [6]	Prospective observational cohort	Evaluate the utility of QFM for analyzing immune function of kidney transplant recipients before and during the first year after transplant and predict infectious complications	36 kidney plus 51 healthy controls and 71 dialysis	Pre‐ and post‐transplant	Pretransplant, 1, 3, 6, and 12 months post‐transplant	1 year	Yes	No	Not reported	Not reported	QFM may have potential for basic pharmacodynamic characterization of immunosuppressive drugs and their combinations, and for assessing loss of global immunocompetence after transplant, but its application to guide drug‐dosing and to predict infectious complications on an individual basis is limited
Mian et al. [24]	Prospective observational cohort	Quantify the degree of immunosuppression and predict subsequent infections using QFM	137 SOT including 50 lung, 44 kidney, 42 liver, 1 small bowel	Post‐transplant	1, 3, and 6 months post‐transplant	1 year	Yes	Yes	No	Not reported	QFM can quantify the level of immunosuppression and predict the risk of subsequent infection episodes in SOT recipients
Solidoro et al. [32]	Prospective observational cohort	Measure QFM at 18 months post‐transplant and correlate with prior infections	15 lung	18 months post‐transplant	Events before 18 months	18 months	Not reported	Yes	Not reported	Not reported	Patients with > 3 infections had lower QFM values at 18 months post‐transplant
Sood et al. [21]	Cross‐sectional	Measure QFM results in healthy, pre‐transplant and post‐transplant individuals	29 who underwent liver transplant plus 30 pre‐transplant patients	Pre‐ and post‐transplant	—	—	Yes	Not reported	Not reported	Not reported	QFM can distinguish between immunosuppressed populations and appears to appropriately increment as time passes, correlating well with progressive reduction in immunosuppression post‐transplant
Sood et al. [26]	Prospective observational cohort	Investigate whether QFM can identify cirrhotic patients at greatest risk of infection	91 cirrhosis, 80 who underwent liver transplant	Pre‐ and post‐transplant	—	Ranging from 2 months to ∼2 years	Yes (pre‐transplant)	Yes	Not reported	Yes (pre‐transplant)	QFM values are lower in patients with cirrhosis, allowing objective determinations of an individual's unique level of immune dysfunction
Sood et al. [23]	Prospective observational cohort	Investigate whether QFM can predict events following transplant including inadequate or excessive immunosuppression, infection, and rejection	75 liver	Pre‐ and post‐transplant	Days 1, 3, and 5, followed by Weeks 1 and 2, and then monthly from 1 to 6 months post‐transplant	1 year	No	Yes	Yes	No	QFM offers the potential for individualization and optimization of immunosuppression

Abbreviations: QFM, QuantiFERON Monitor; SOT, solid organ transplant.

A variety of different clinical outcomes were assessed. Infection was the most common outcome measured and was reported in 8 studies. Organ rejection and mortality were assessed in 3 and 2 studies each, respectively. Eight studies investigated relationships between immunosuppression regimens, dosing of immunosuppressive therapies, and QFM results.

#### 3.2.2 Relationships Between QFM Values and Infection Outcomes

The definition of infection varied considerably in the 8 studies that investigated infection as an outcome and included both clinical and laboratory classifications (Table 2). Most studies used a composite endpoint of any infection [6, 14, 23, 24, 26, 31, 32]. Other infection outcomes included viral, bacterial, and opportunistic and/or serious infections (Table 2). Full definitions of reported infection measures can be found in Table S2.

**TABLE 2:** Outcomes reported in the 8 identified studies that assessed QuantiFERON Monitor as a predictor of infection in solid organ transplant recipients.

Study	Type of infection	QFM threshold for infection (IU/mL)		OR (95% CI)	HR (95% CI)^a^	Infection outcomes	Study conclusions
Fernandez‐Ruiz et al. [31]	Any infection	15		—	—	No significant differences in infections overall in those with low values. Patients with low QFM values at 2 weeks were more likely to develop bacterial infection and with low QFM values at 4 weeks were more likely to develop opportunistic infection than those with high QFM values	The discriminative ability of QFM was low overall. QFM has some potential utility to predict early bacterial and opportunistic infections. Performance was improved with alternative cutoff values
	Bacterial infection (Week 2)	7.9		—	—		
	Opportunistic infection (Week 4)	47.3		—	—		
Gardiner et al. [14]	Any infection (bacterial, fungal, viral, mycobacteria and polymicrobial including CMV viremia and mold infections)	—	—	—	—	During early posttransplant, QFM was unable to reliably distinguish between patients with/without infections due to low values with substantial overlap. However, beyond 3 months, patients with lower QFM values were more likely to develop serious opportunistic infections	QFM may be able to identify overly immunosuppressed patients as low values were associated with infection beyond 3 months posttransplant
	Serious infection	2–6 weeks	2	—	1.41 (0.52–3.78)		
		6 weeks–3 months	6	—	1.03 (0.39–2.71)		
		3–6 months	16	—	2.22 (0.97–5.09)		
		6–12 months	60	—	2.90 (1.34–6.25)		
	Opportunistic infection	2–6 weeks	4	—	2.28 (0.56–9.33)		
		6 weeks–3 months	6	—	0.49 (0.05–4.67)		
		3–6 months	48	—	2.53 (0.80–7.95)		
		6–12 months	92	—	1.68 (0.84–3.37)		
	Serious opportunistic infection	2–6 weeks	2	—	2.77 (0.46–16.59)		
		6 weeks–3 months	194	—	0.06 (0–0.92)		
		3–6 months	10	—	6.38 (1.37–29.66)		
		6–12 months	60	—	3.25 (1.11–9.49)		
Margeta et al. [19]	Bacterial infection	—		—	—	Kidney transplant recipients presenting with a bacterial infection had significantly lower QFM values compared with stable kidney transplant recipients (*p* = 0.04). Patients in the stable kidney transplant recipients group also had a numerically higher QFM values than patients in the infection group, but it was not statistically significant (*p* = 0.24)	QFM can be useful for guiding immunosuppression dosing in kidney transplant patients as recipients with bacterial infection had lower QFM values compared with stable kidney transplant recipients
	Viral infection						
Marx et al. [6]	Any infection (bacterial, fungal, viral)	—		—	—	QFM levels prior to transplant were significantly higher in patients with infections in the first month. QFM values at later timepoints tended to be lower in patients with subsequent infection, although this did not reach statistical significance	Although the QFM test may have potential for assessing immunocompetence after transplant, it is unable to predict infections on an individual basis
Mian et al. [24]	Any infection including bacterial infections, fungal infections, CMV viremia, BK viremia, EBV, and opportunistic infections	Month 1 (between 1 and 3 months)	10	2.26 (0.97–5.25)	—	Low QFM values were associated with increased risk of infection, particularly opportunistic infections at 3 and 6 months (*p* = 0.024 and *p* = 0.014, respectively)	The QFM test can predict the risk of subsequent infections in organ transplant recipients
		Month 3 (between 3 and 6 months)	10	3.88 (1.55–9.70)	—		
		Month 6 (between 6 and 12 months)	10	2.78 (1.09–7.09)	—		
Solidoro et al. [32]	> 3 episodes of any infection	89.5		—	—	Patients with > 3 infections prior had lower QFM values at 18 months than those with ≤ 3 infections	Low QFM values were associated with infection
Sood et al. [26]	Any infection	214		—	4.1	Low QFM values were associated with increased susceptibility to infection	The QFM test can be used to quantify the immunosuppressed status of cirrhotic patients which can then be used to predict infection risk
Sood et al. [23]	Infection	Week 1–Month 1	1.30	—	—	Low QFM values at Week 1 were significantly associated with the risk of infection (AUROC, 0.74; *p* = 0.008)	The QFM test can be used to predict risk of infection as low QFM values indicate oversuppression and are associated with infection
	Opportunistic	Week 1	0.33				

Abbreviations: AUROC, area under the receiver operating characteristic curve; EBV, Epstein–Barr virus; CI, confidence interval; CMV, cytomegalovirus; HR, hazard ratio; OR, odds ratio; QFM, QuantiFERON Monitor.

^a^ For patients testing below versus above stated threshold value.

Seven of the 8 studies that reported infection outcomes found an association between low QFM values and higher rates of infection, although in some cases associations were only identified in specific subgroups (e.g., for certain infection types; Table 2) [14, 19, 23, 24, 26, 31, 32]. Lower QFM values were reported to be significantly associated with at least 1 episode of infection, particularly opportunistic infections, at 3 and 6 months posttransplant, in a study including lung, liver, and kidney recipients [24]. A significant correlation between low QFM values at 1 week posttransplant and increased risk of early infection was also reported in liver transplant recipients [23]. In cirrhotic patients pretransplant, low QFM values were associated with pretransplant infection risk [26]. Similarly, lung transplant patients with lower QFM values were more likely to develop serious opportunistic infections, including bacterial, viral, and fungal infections, than those with higher values at certain time periods and cutoff thresholds [14]. Due to significant overlap in QFM values between recipients who did and did not develop infections, the QFM test was determined to be unreliable for identifying patients with infection in the first 3 months post‐lung transplant [14]. Solidoro et al. identified lower QFM values at 18 months after lung transplant in those who had experienced > 3 infections compared with those who experienced ≤ 3 infections (69 ± 22 vs. 381 ± 105 IU/mL, *p* = 0.033), although the sample size was small (*n* = 15) [32]. In another study, kidney transplant recipients with bacterial infections had significantly lower QFM values than those without, although no statistical difference was reported for infections overall [19].

In contrast to these studies, Fernandez‐Ruiz et al. explored QFM in 126 kidney recipients and found that, overall, patients with infections had similar QFM values to those without; however, when stratified by infection type, patients with early bacterial or opportunistic infections had lower QFM values than those without at 2 and 4 weeks, respectively [31]. Similarly, an analysis of 36 kidney transplant recipients failed to identify a clear association between QFM values at 3 and 6 months posttransplant and risk of any infection, including bacterial, fungal, and viral infections [6]. While QFM values were lower in recipients with bacterial and viral infections than those without at both 3 and 6 months posttransplant, respectively, the authors concluded that the QFM test had limited value for predicting infection due to the high interindividual variability in QFM values. Statistical power was limited by the small sample size [6].

Multiple QFM threshold values for infections were reported across the studies, ranging from 0.33 to 214 IU/mL [14, 23, 24, 26, 31, 32]. In addition, the ranges and thresholds used to define low and high QFM values varied considerably between studies, depending on the timepoint posttransplant, the patient population, and the outcomes assessed (Table 2). A threshold of QFM 1.3 IU/mL was associated with the highest infection risk and minimal rejection risk in liver transplant recipients [23]. A lower week 1 threshold of 0.33 IU/mL for opportunistic infections was also reported. In another study, lung transplant recipients with QFM values < 10 and < 60 IU/mL were more likely to develop a serious opportunistic infection between 3–6 months and 6–12 months posttransplant, respectively, compared with those testing above these thresholds [14]. Similarly, using a threshold value of 10 IU/mL also significantly increased the likelihood of infection at 1–3, 3–6, and 6–12 months posttransplant in a study including mixed SOT populations [24]. In kidney transplant recipients, a QFM threshold of 7.9 IU/mL at 2 weeks had improved specificity for bacterial infection and 47.3 IU/mL at 4 weeks had improved sensitivity for opportunistic infections compared with manufacturer‐recommended cutoffs [31]. In addition, a threshold of 214 IU/mL in patients pretransplant was also shown to correlate with increased risk for infection pre‐liver transplant [26]. Performance characteristics including sensitivity, specificity, and positive/negative predictive values (PPV/NPV) were reported in 4 studies and ranged widely (Table S3). Across all studies assessing different time periods and infection outcomes in SOT recipients, the median calculated optimal cutoff was 9 IU/mL (IQR 5–57). Median (IQR) sensitivity, specificity, PPV, and NPV were 65% (55%–75%), 60% (54%–67%), 31% (25%–51%), and 84% (70%–92%), respectively. AUCs were modest, with a median of 60% (55%–67%).

#### 3.2.3 Relationships Between QFM Values and Organ Rejection

Three studies assessed organ rejection as a clinical outcome (Table 3) [14, 23, 24]. High QFM values at 1 week post‐liver transplant were significantly associated with the risk of rejection, potentially indicating under‐immunosuppression [23]. An increased risk of rejection was reported when a QFM threshold value of 4.49 IU/mL was exceeded between 1 week and 1 month posttransplant. Conversely, 2 studies found no clear relationship between rejection and QFM values measured at various timepoints throughout the first year posttransplant in liver, kidney, and lung recipients, in part due to the low number of rejection events [14, 24]. As a result, these studies were unable to reliably propose QFM threshold values for rejection [14, 24].

**TABLE 3:** Outcomes reported in the 3 identified studies that assessed QuantiFERON Monitor as a predictor of rejection in solid organ transplant recipients.

Study	Definition of rejection	QFM threshold for rejection (IU/mL)		OR (95% CI)^a^	Rejection outcomes	Study conclusions
Gardiner et al. [14]	Acute cellular rejection according to the International Society of Heart and Lung Transplantation scoring system	2–6 weeks	2	—	There was a very low number of rejection events within each time period, and no clear relationship was identified between QFM values and rejection	QFM was not useful as a tool for the prediction of rejection in lung transplant recipients
		6 weeks–3 months	2	—		
		3–6 months	266	—		
		6–12 months	30	—		
	Rejection requiring treatment with high‐dose steroids	2–6 weeks	2	—		
		6 weeks–3 months	162	—		
		3–6 months	266	—		
		6–12 months	30	—		
Mian et al. [24]	Biopsy‐proven rejection	—	—	—	No significant differences were noted between QFM levels in patients who developed rejection versus those who had no rejection episode	QFM was not useful as a tool for the prediction of subsequent rejection episodes in transplant recipients
Sood et al. [23]	Biopsy‐proven acute rejection, with histology confirmed by a specialist liver transplant pathologist and treatment commenced by the transplant physician caring for the patient who was blinded to QFM results	1 week–1 month	4.49	4.75	Elevated QFM values at week 1 were significantly associated with rejection within the first month following transplant (AUROC, 0.77; *p =* 0.002)	QFM could be used to predict risk of rejection as high values indicate undersuppression and were associated with rejection

Abbreviations: AUROC, area under the receiver operating characteristic curve; CI, confidence interval; OR, odds ratio; QFM, QuantiFERON Monitor.

^a^ For patients testing below versus above stated QFM threshold value.

#### 3.2.4 Relationships Between QFM Values and Mortality

Two studies assessed the relationship between QFM values and mortality (Table S4) [23, 26]. Low QFM values were associated with mortality in cirrhotic patients awaiting liver transplant [26] but not following liver transplant [23]. Incidence of death was low overall in all 3 studies (3.3%–4.0%).

#### 3.2.5 Relationships Between QFM Values and Immunosuppression Regimens, Including Types and Doses of Immunosuppressive Therapy

Seven studies explored the relationship between immunosuppression regimens and QFM values [6, 14, 19, 21, 23, 24, 31]. Notably, the types and doses of immunosuppressive therapies used in each study differed, with prednisolone and tacrolimus being the most common.

Six studies reported clear associations between specific immunosuppressive regimens and QFM values [6, 14, 19, 21, 24, 31]. Immunosuppressive therapies, treatment doses, dose intensity, and statistical analyses used to assess the relationships between immunosuppression regimens and QFM values in these studies are outlined in Table S5. Prednisolone and tacrolimus doses were inversely associated with QFM values in lung transplant recipients, noting that the association was stronger with prednisolone [14]. In kidney recipients, tacrolimus levels were negatively correlated with QFM values, although not statistically significant [19], and cyclosporine A, methylprednisolone, and tacrolimus demonstrated strong dose‐dependent relationships with QFM values in vitro, with reduced QFM values when immunosuppressive therapies were combined [6]. Prednisone and mycophenolate also showed a negative correlation with QFM values at all timepoints and at 1 and 6 months posttransplant, respectively, in another study including mixed SOT populations [24]. In this group, tacrolimus demonstrated a weak correlation that did not reach statistical significance. In contrast, tacrolimus and cyclosporine levels in liver transplant recipients and tacrolimus levels in kidney transplant recipients were not significantly associated with QFM values, although kidney recipients who received thymoglobulin induction had lower values in the first month than at subsequent timepoints [21, 31].

### 3.3 HCT Recipients

Only 4 publications reporting on the relationship between QFM values and clinical outcomes in a total of 180 HCT patients were identified (Tables S3, S6, and S7). Reported clinical outcomes included infection, GvHD, and mortality. Although much smaller in size than the SOT studies, most reported an association between QFM values and one or more clinical outcomes. In one study, low QFM values were associated with both low (CMV DNA ≥ 500 IU/mL) and high‐level CMV infection (CMV DNA ≥ 5000 IU/mL), with a threshold of 86.95 IU/mL predictive of both at 4 weeks post‐HCT [25]. Similarly, a significant association between low QFM values and CMV infection was reported in another study, albeit with methodological limitations, which also found a significant negative association between QFM results and mortality, and a nonsignificant positive association with GvHD incidence [22]. No significant associations were found by the same authors in pediatric HCT recipients [33]. Notably, Douglas et al. reported that QFM values were significantly lower in those with active infections compared with those without during the neutropenic period prior to engraftment [13]. In addition, QFM values were significantly lower in those with CMV viral load > 1000 copies/mL compared with those with a load < 1000 copies/mL during the first 180 days. Due to the low number of reported events, however, the study was unable to establish an association between QFM values and infection or GvHD post‐HCT.

## 4 Discussion

Transplant recipients experience a high burden of infections, particularly throughout the first year, which contribute to significant morbidity and mortality [13, 34, 35, 36, 37]. Global immune biomarker assays such as the QFM test have the potential to measure the net state of immunosuppression and identify overly immunosuppressed patients who may be at increased risk for infectious complications, and those with insufficient immunosuppression who may be at increased risk for rejection [14]. In this scoping review, we have summarized the available evidence on the use of the QFM test to predict clinical outcomes, focusing primarily on SOT recipients. The most commonly evaluated outcome in SOT recipients was infection, and the majority of studies found that lower QFM values were associated with an increased risk of infection [14, 19, 23, 24, 26, 31, 32]. Seven studies assessed the relationship between immunosuppression regimens and QFM values, with most reporting that higher doses correlated with lower QFM values in SOT recipients [6, 14, 19, 21, 24, 31]. Studies evaluating relationships with organ rejection and mortality failed to demonstrate consistent associations in SOT populations, possibly due to the low number of events reported which hindered the ability to draw meaningful conclusions [14, 23, 24, 26].

There was a high degree of heterogeneity in the design of the SOT studies identified, with variation in the transplanted organ population, immunosuppression and antimicrobial prophylactic regimens used, the clinical outcomes assessed and their definitions, the QFM thresholds identified, and the time points evaluated. These differences often prevented meaningful comparison of the utility of the QFM test when evaluating relevant clinical outcomes across the studies. For example, it is possible that lung transplant recipients may be more immunosuppressed, have lower QFM values and experience more infections than kidney recipients, but it is difficult to determine this reliably from the data available. While only a small number of studies provided details of performance characteristics, overall sensitivity, specificity, and PPVs and AUCs were modest. NPVs were a little higher, suggesting that patients with higher QFM values may be less likely to develop infections.

In addition, there was significant variability in the assessment and definitions of infections, including several studies examining bacterial, viral, fungal, and opportunistic infections in different ways. A more standardized approach to assessing and reporting infection outcomes would be helpful to improve comparability across studies [38]. It seems plausible that global immune biomarker assays such as QFM would be more likely to predict infections related to immunosuppression such as CMV or invasive fungal infections rather than community acquired respiratory viruses or line‐associated bacteremias, for example, but few studies have specifically examined these less common outcomes in a clear and consistent way.

The optimal timing for use of these assays also requires further consideration. In the first month posttransplant, recipients are highly immunosuppressed and infections are probably more likely to be related to perioperative, donor and nosocomial factors rather than the net state of immunosuppression. Beyond the first year, opportunistic infections are less common. There are important differences between cell‐ and antibody‐mediated rejection, with factors beyond the level of immunosuppression, such as the donor‐recipient match and development of donor‐specific antibodies, also being important. This may explain why QFM values did not seem to be associated with rejection, a relatively infrequent event, in many studies.

Because many decisions clinicians are faced with are binary, having well‐established thresholds to inform actions is important. However, defining meaningful thresholds, or “cutoffs,” for global immune biomarker assays, such as the QFM test, to guide clinical decision‐making is a major challenge. Converting continuous variables into binary categories results in loss of nuanced information and oversimplification which can lead to misclassification, particularly for values near the threshold. This is exemplified by the trade‐offs seen between sensitivity and specificity and leads to false positive and false negative results. There are also challenges with generalizability, where cutoffs derived in one population may not generalize well to other settings or patient groups. In the studies identified, the QFM threshold values and ranges evaluated for the prediction of infection risk varied widely between the studies, ranging between 0.33 and 214 IU/mL [14, 23, 24, 26, 31, 32]. Values varied depending on the timing of blood sample collection and the transplant population. It remains unclear how best to apply these assays to an individual patient, and whether interpretation of results is influenced by factors such as the transplanted organ type, nature of immunosuppression, and time posttransplant, or if a single threshold could eventually be widely applied across multiple patient populations. Further studies are needed to evaluate the quantitative threshold value for predicting infection and to compare the optimal timepoints or timeframes for posttransplant monitoring in different SOT populations.

Our review focused primarily on SOT recipients, as this group has more published data on the use of the QFM test. Due to immunological differences between the two populations, immune biomarkers need to be considered and applied differently in HCT recipients [39]. The 3 studies reporting on GvHD, an important outcome, found no association with QFM values [13, 22, 25]. All 4 of the HCT studies identified reported on infection as an outcome, and 3 suggested that the QFM test may have predictive utility for infection and/or mortality in HCT recipients [13, 22, 25]. Further large prospective studies evaluating the use of the QFM test are needed to determine its potential to predict risk in the HCT setting. Outside of transplant, QFM has been explored for predicting the risk of infection in patients with end‐stage kidney disease [40] and for understanding immune function in patients with multiple sclerosis [41, 42].

This is the first comprehensive review of the available literature on the use of the QFM test to determine the net state of immunosuppression and predict clinical outcomes in SOT and HCT recipients. The studies included were evaluated in detail and included different transplant types and clinical outcomes. There are some limitations that should be considered when interpreting our results. We did not conduct a quantitative analysis, and there was substantial heterogeneity among the factors reported that impeded a formal meta‐analysis. The review was also limited by the number and quality of observational studies available. Several studies had methodological limitations, were cross‐sectional in nature or had small sample sizes and low numbers of clinical events, limiting statistical power and the ability to assess QFM as a true predictor. This was particularly problematic for rejection outcomes, which were rare during the early posttransplant period. Similarly, deaths were rare in the first year posttransplant and studies may need to look beyond this time period to fully assess the relationship with mortality. Few studies reported performance characteristics such as area under the receiver operating characteristic curve, sensitivity, specificity, and predictive values, making it difficult to report on diagnostic accuracy. Finally, how QFM performs in comparison to, or in combination with, other global immune biomarkers such as the absolute lymphocyte count, lymphocyte subsets, immunoglobulin levels, torque teno virus, and others, as well as transplant‐specific diagnostics using cell‐free DNA to predict rejection, allograft injury and titrate immunosuppression is unclear and requires further study. This will help determine the optimal timing and use, perhaps in combination, of these biomarkers in routine transplant care.

In conclusion, global immune biomarkers assays, such as the QFM test, have the potential to increase our ability to measure the net state of immunosuppression and to help identify patients who are excessively or insufficiently immunosuppressed posttransplant and at increased risk for related complications. Existing studies assessing the use of QFM in transplant recipients are limited and heterogeneous, but have identified associations between low QFM values, higher levels of immunosuppression, and infections. It is not yet clear what these results mean for an individual patient, or how to integrate and implement these assays into clinical practice. More studies are needed, including larger prospective studies with training and validation cohorts, as well as interventional trials, to further examine the role of QFM and confirm its predictive utility and value in the management of SOT and HCT recipients. These studies should aim to determine the optimal time points for collecting samples posttransplant, develop meaningful thresholds to guide clinical decision‐making, examine cost‐effectiveness and explore the safety and efficacy of modifying immunosuppression for an individual patient guided by QFM results. This approach has the potential to improve our ability to predict and prevent infections in transplant recipients by developing a precision medicine method to personalize immunosuppression dosing regimens. It could also inform other interventions such as enhanced clinical monitoring and prophylactic antimicrobials, improving quality of life and survival.

## Author Contributions

**Bradley J. Gardiner**: investigation, writing – original draft, formal analysis, data curation. **Roy F. Chemaly**: supervision, writing – review and editing. **Vladyslav Nikolayevskyy**: conceptualization, funding acquisition, writing – review and editing, methodology. **Riccardo Alagna**: conceptualization, funding acquisition, writing – review and editing, methodology. **Davide Manissero**: conceptualization, funding acquisition, methodology, writing – review and editing. **Camille N. Kotton**: writing – review and editing; supervision.

## Conflicts of Interest

Bradley J. Gardiner has received honoraria from QIAGEN and Takeda Pharmaceuticals. Roy F. Chemaly has served as a consultant, scientific advisor, or speaker for ADMA Biologics, AiCuris, Ansun Biopharma, Astellas, Eurofins‐Viracor, Gilead, InflaRx, Karius, Merck/MSD, Moderna, Oxford Immunotec, Partner Therapeutics, Pfizer, Roche (Genentech), Shionogi, Takeda Pharmaceuticals, and Tether. Roy F. Chemaly also received research grants paid to his institution from AiCuris, Ansun Biopharma, Eurofins‐Viracor, Freestyle, Karius, Merck, Oxford Immunotec, Roche (Genentech), and Takeda Pharmaceuticals. Vladyslav Nikolayevskyy is an employee of QIAGEN. Riccardo Alagna is an employee of QIAGEN. Davide Manissero was an employee of QIAGEN. Camille N. Kotton has received honoraria from Abbott, BioHope, Biotest, Merck, QIAGEN, Roche Diagnostics, and Takeda Pharmaceuticals. While several authors are employees of Qiagen and the work was sponsored by the company, the literature review, analysis, and manuscript writing were performed objectively, and these affiliations did not influence or bias the conclusions reached.

## Supporting information




**Table S1**: Complete search strategies for database searches.
**Table S2**: Definition of infection measures reported in the 8 identified studies that assessed QuantiFERON Monitor as a predictor of infection in solid organ transplant recipients.
**Table S3**: Details of performance characteristics from the 5 studies that reported this data, including timepoint, outcome and cutoff assessed.
**Table S4**: Outcomes reported in the 2 identified studies that assessed QuantiFERON Monitor as a predictor of mortality in solid organ transplant recipients.
**Table S5**: Key findings from the 6 identified studies that evaluated relationships between immunosuppression and QuantiFERON Monitor results in solid organ transplant recipients.
**Table S6**: Overall findings of the 4 identified studies that evaluated QuantiFERON Monitor in HCT recipients.
**Table S7**: Clinical outcomes and results reported in the 4 identified studies that evaluated QuantiFERON Monitor in HCT recipients.

## Data Availability

Data sharing is not applicable to this article as no datasets were generated or analyzed.
