# Supplementary material for: Use of the QuantiFERON Monitor Assay to Predict Clinical Outcomes in Solid Organ and Hematopoietic Cell Transplant Recipients: A Scoping Review
Source: Transpl Infect Dis. 2025 Jul 1;27(4):e70074. doi: 10.1111/tid.70074 (PMC12416346; doi:10.1111/tid.70074)
Supplement: Supplementary file 1 — Table S1: Complete search strategies for database searches. Table S2: Definition of infection measures reported in the 8 identified studies that assessed QuantiFERON Monitor as a predictor of infection in solid organ transplant recipients. Table S3: Details of performance characteristics from the 5 studies that reported this data, including timepoint, outcome and cutoff assessed. Table S4: Outcomes reported in the 2 identified studies that assessed QuantiFERON Monitor as a predictor of mortality in solid organ transplant recipients. Table S5: Key findings from the 6 identified studies that evaluated relationships between immunosuppression and QuantiFERON Monitor results in solid organ transplant recipients. Table S6: Overall findings of the 4 identified studies that evaluated QuantiFERON Monitor in HCT recipients. Table S7: Clinical outcomes and results reported in the 4 identified studies that evaluated QuantiFERON Monitor in HCT recipients. [file TID-27-e70074-s001.docx]

# SUPPLEMENTAL DIGITAL CONTENT

# Use of the QuantiFERON Monitor^®^ Assay to Predict Clinical Outcomes in Solid Organ and Hematopoietic Cell Transplant Recipients: A Scoping Review

Bradley J. Gardiner, MBBS, PhD, Roy F. Chemaly, MD, Vladyslav Nikolayevskyy, PhD, Riccardo Alagna, MSc, Davide Manissero, MD, and Camille N. Kotton, MD

#### **Table S1. Complete search strategies for database searches**

| **Database** | **Search number** | **Search strategy/query** | **Results** |
| --- | --- | --- | --- |
| **PubMed** | #1 | "QuantiFERON Monitor"[tiab:~2] OR "QuantiFERON-Monitor”[all] OR "QF monitor"[tiab:~2] OR "QF-monitor"[tiab:~2] | 19 |
| **Embase** | #1 | ('quantiferon'/exp OR quantiferon) AND adj2 AND ('monitor'/exp OR monitor) OR 'quantiferon-monitor' OR (qf AND adj2 AND ('monitor'/exp OR monitor)) OR 'qf-monitor' | 38 |
|  | #2 | #1 AND [embase]/lim | 37 |
| **Cochrane CENTRAL** | #1 | (QuantiFERON NEAR/2 Monitor) OR ("QuantiFERON-Monitor") OR (QF NEAR/2 monitor) OR ("QF-monitor") | 4 |
|  | #2 | #1 in Trials | 4 |

#### **Table S2. Definition of infection measures reported in the 8 identified studies that assessed QuantiFERON^®^ Monitor as a predictor of infection in solid organ transplant recipients**

| **Study** | **Type of infection** | **Definition of infection** |
| --- | --- | --- |
| Fernandez-Ruiz 2024^1^ | Bacterial infection  Opportunistic infection | Microbiologic findings in association with a compatible clinical syndrome, using definitions proposed by the Centers for Disease Control and Prevention’s National Healthcare Safety Network (CDC/NHSN)^2^  Opportunistic infections included tuberculosis, listeriosis, herpes simplex & varicella zoster viruses, cytomegalovirus, BK nephropathy, invasive fungal disease, Pneumocystis pneumonia, toxoplasmosis and visceral leishmaniasis |
| Gardiner 2021^3^ | Any infection (bacterial, fungal, viral, mycobacteria, and polymicrobial including CMV viremia and mold infections) | Defined as per the pathogen detected by microbiological testing. Infections were classified as ‘polymicrobial’ if more than one potential pathogen was isolated in appropriately collected cultures and the clinical infection syndrome could not be attributed to one pathogen alone (excluding same class pathogens) |
|  | Serious infection | Defined as infection requiring hospitalization or if the individual was already hospitalized for another reason and the infection resulted in prolongation of the admission |
|  | Opportunistic infection | Defined as infections related to immunosuppression that are unlikely to impact healthy individual |
|  | Serious opportunistic infection | Defined as infections related to immunosuppression that are unlikely to impact healthy individuals, and that require hospitalization or if the individual was already hospitalized for another reason and the infection resulted in prolongation of the admission |
| Margeta 2020^4^ | Bacterial infection | Defined as elevated CRP (>20 mg/dL), clinical and laboratory signs and symptoms (eg, positive urine/blood cultures, fever, chest x-ray suggestive of infection), and prescription of antimicrobial drugs by the transplant physician |
|  | Viral infection | CMV active disease and infection were defined by the American Society of Transplantation criteria.^5^ Both the presumptive and definitive BK viremia nephropathy diagnosis were used as inclusion criteria. Other infections were defined as elevated CRP (>20 mg/dL), clinical and laboratory signs and symptoms (eg, positive urine/blood cultures, fever, chest x-ray suggestive of infection), and prescription of antimicrobial drugs by the transplant physician |
| Marx 2020^6^ | Any infection (bacterial, fungal, viral) | Defined microbiologically or based on clinical diagnosis and/or response to treatment |
| Mian 2018^7^ | Any infection | Defined according to the American Society of Transplantation Guidelines |
|  | Bacterial infection | Defined according to the American Society of Transplantation Guidelines |
|  | Fungal infections | Defined as ‘proven’ or ‘probable’ infection as per the American Society of Transplantation Guidelines. Infections were also defined based on European Organization for Research and Treatment of Cancer/Invasive Fungal Infections Cooperative Group and National Institute of Allergy and Infectious Diseases Mycoses Study Group definitions |
|  | CMV viremia and BK viremia | Defined according to the American Society of transplantation Guidelines |
|  | EBV | Defined according to the American Society of transplantation Guidelines |
|  | Opportunistic infections | Pre-defined and includes any viral, fungal, or mycobacterial infection |
| Solidoro 2025^8^ | >3 infections of any type | Total number of infections including pulmonary and extra-pulmonary, including bacterial, fungal, CMV and other viral infections. |
| Sood 2016^9^ | Any infection | ‘Probable’ or ‘definite’ infection adjusted from the pre-defined criteria stated in The International Sepsis Forum Consensus Conference on ‘Definitions of Infection in the Intensive Care Unit’ |
| Sood 2017^10^ | Infection | As per predefined criteria of ‘probable’ or ‘definite’ infection adjusted from the international sepsis forum consensus conference on ‘Definitions of Infection in the Intensive Care Unit’. CMV reactivation without disease was not defined as an infection |
|  | Opportunistic | – |

EBV, Epstein-Barr virus; CMV, cytomegalovirus; CRP, C-reactive protein; SOT, solid organ transplant.

**Table S3. Details of performance characteristics from the 5 studies that reported this data, including timepoint, outcome and cutoff assessed**

| **Study** | **Timepoint** | **Outcome** | **Cutoff** | **Sensitivity** | **Specificity** | **PPV** | **NPV** | **AU-ROC** |
| --- | --- | --- | --- | --- | --- | --- | --- | --- |
| Sood S, et al. Liver Transpl 2017;23:487–97 | 1 week-1 month | Infection | 1.3 | 63% | 71% | 37% | 88% | 74% |
|  |  |  | 4.49 | 100% | 28% | NR | NR | 74% |
|  |  | Biopsy proven rejection | 1.3 | 93% | 52% | NR | NR | 77% |
|  |  |  | 2.61 | 64% | 72% | 41% | 87% | 77% |
|  |  |  | 4.49 | 50% | 85% | 50% | 85% | 77% |
|  |  | Opportunistic infection | 0.33 | 50% | 94% | NR | NR | 72% |
| Gardiner BJ, et al. Transpl Infect Dis 2021;23:e13550 | 2-6 weeks | Any infection | 6 | 43% | 67% | 45% | 66% | 51% |
|  |  |  | 15 | 80% | 20% | 37% | 62% | NR |
|  |  | Opportunistic infection | 4 | 67% | 50% | 15% | 92% | 54% |
|  |  |  | 15 | 21% | 89% | 13% | 94% | NR |
|  |  | Serious infection | 2 | 56% | 57% | 25% | 84% | 58% |
|  |  |  | 15 | 24% | 94% | 24% | 94% | NR |
|  |  | Serious OI | 2 | 80% | 57% | 11% | 98% | 64% |
|  |  |  | 15 | 22% | 100% | 8% | 100% | NR |
|  |  | Any rejection | 2 | 60% | 65% | 10% | 96% | 50% |
|  |  |  | 15 | 20% | 80% | 6% | 94% | NR |
|  |  | Steroid rejection | 2 | 75% | 65% | 10% | 98% | 53% |
|  |  |  | 15 | 20% | 75% | 5% | 94% | NR |
|  | 6 weeks-3 months | Any infection | 6 | 55% | 60% | 50% | 64% | 52% |
|  |  |  | 15 | 42% | 64% | 45% | 61% | NR |
|  |  | Opportunistic infection | 6 | 75% | 55% | 8% | 98% | 46% |
|  |  |  | 15 | 41% | 75% | 6% | 97% | NR |
|  |  | Serious infection | 6 | 53% | 56% | 25% | 81% | 47% |
|  |  |  | 15 | 39% | 59% | 21% | 77% | NR |
|  |  | Serious OI | 194 | 50% | 96% | 25% | 99% | 70% |
|  |  |  | 15 | 61% | 50% | 3% | 98% | NR |
|  |  | Any rejection | 2 | 60% | 85% | 21% | 97% | 58% |
|  |  |  | 15 | 40% | 60% | 6% | 94% | NR |
|  |  | Steroid rejection | 162 | 50% | 93% | 29% | 97% | 49% |
|  |  |  | 15 | 61% | 50% | 6% | 96% | NR |
|  | 3-6 months | Any infection | 48 | 54% | 52% | 61% | 45% | 47% |
|  |  |  | 15 | 55% | 48% | 59% | 43% | NR |
|  |  | Opportunistic infection | 48 | 75% | 54% | 29% | 89% | 60% |
|  |  |  | 15 | 57% | 62% | 27% | 86% | NR |
|  |  | Serious infection | 16 | 62% | 60% | 41% | 79% | 56% |
|  |  |  | 15 | 60% | 62% | 41% | 79% | NR |
|  |  | Serious OI | 10 | 82% | 62% | 26% | 95% | 64% |
|  |  |  | 15 | 59% | 82% | 24% | 95% | NR |
|  |  | Any rejection | 266 | 50% | 92% | 33% | 96% | 49% |
|  |  |  | 15 | 47% | 50% | 7% | 92% | NR |
|  |  | Steroid rejection | 266 | 50% | 92% | 33% | 96% | 49% |
|  |  |  | 15 | 47% | 50% | 7% | 92% | NR |
|  | 6-12 months | Any infection | 114 | 67% | 59% | 85% | 33% | 61% |
|  |  |  | 15 | 88% | 30% | 90% | 26% | NR |
|  |  | Opportunistic infection | 92 | 64% | 49% | 52% | 61% | 56% |
|  |  |  | 15 | 76% | 28% | 50% | 54% | NR |
|  |  | Serious infection | 60 | 63% | 66% | 54% | 74% | 65% |
|  |  |  | 15 | 81% | 37% | 55% | 67% | NR |
|  |  | Serious OI | 60 | 65% | 60% | 31% | 86% | 59% |
|  |  |  | 15 | 75% | 29% | 25% | 79% | NR |
|  |  | Any rejection | 30 | 67% | 75% | 26% | 94% | 69% |
|  |  |  | 15 | 76% | 44% | 20% | 91% | NR |
|  |  | Steroid rejection | 30 | 67% | 75% | 26% | 94% | 69% |
|  |  |  | 15 | 76% | 44% | 20% | 91% | NR |
| Yoon, E., et al. Transpl Infect Dis: e14328. | 4-24 weeks | Any CMV infection | 463 | 85% | 78% | NR | NR | NR |
|  |  | High-level CMV infection | 86.95 | 89% | 47% | NR | NR | NR |
| Fernandez-Ruiz et al. Transpl Int 2024; 37:13551 | 2-52 weeks | Bacterial infection | 15 | 75% | 52% | 51% | 76% | 61% |
|  |  |  | 7.9 | 68% | 67% | 58% | 76% | 67% |
|  | 1-12 months | Opportunistic infection | 15 | 55% | 70% | 32% | 86% | 67% |
|  |  |  | 47.3 | 82% | 51% | 30% | 92% | 57% |
| Solidoro P et al. Microorganisms 2025 13(2) | 18-36 months | Infection | 89.5 | 78% | 77% | NR | NR | 81% |

AU-ROC, area under the receiver operating characteristic curve. CMV, cytomegalovirus. OI, opportunistic infection. NPV, negative predictive value. NR, not reported. PPV, positive predictive value.

#### **Table S4. Outcomes reported in the 2 identified studies that assessed QuantiFERON^®^ Monitor as a predictor of mortality in solid organ transplant recipients**

| **Study** | **Deaths/population analyzed (%)** | **QFM threshold for mortality (IU/mL)** | **HR*** | **Mortality outcome** | **Study conclusions** |
| --- | --- | --- | --- | --- | --- |
| Sood 2016^9^ | 3/91 (3.3%) | <30 | 56.6 | A very low QFM <30 IU/mL was significantly associated (*P* = 0.003) with death in 3 patients who died while awaiting transplant | Patients who died pre-transplant had a significantly lower QFM value than those who did not |
| Sood 2017^10^ | 3/75 (4.0%) | – | – | Three deaths occurred during the study period, resulting in a 12-mo survival of 96.0% | No association was found between pre- or post-transplant QFM values and mortality |

*For patients testing below versus above stated QFM threshold value.

HR, hazard ratio; QFM, QuantiFERON^®^ Monitor; SOT solid organ transplant.

#### **Table S5. Key findings from the 6 identified studies that evaluated relationships between immunosuppression and QuantiFERON^®^ Monitor results in solid organ transplant recipients**

| **Study** | **Description of statistical analysis** | **Immunosuppressive agent** | **Values (95% CI)** | ***P*** | **Treatment outcome** | **Study conclusions** |
| --- | --- | --- | --- | --- | --- | --- |
| Fernandez-Ruiz 2024^1^ | QFM values compared using Wilcoxon test (continuous values) or McNemar test (categorical). Correlations assessed with Pearson’s r | Induction thymoglobulin | Week 2 low response, n (%): 39 (59.1) | 0.001 | Patients receiving thymoglobulin induction had lower QFM values at 2 and 4 weeks post-transplant than those who didn’t receive thymoglobulin. Weak inverse correlation was identified between tacrolimus trough levels and QFM results at 6 months but not earlier | The performance of QFM decreases late post-transplant, once the amount of immunosuppression has been stabilized in most recipients |
|  |  |  | Week 2 moderate-high response, n (%): 13 (28.3) |  |  |  |
|  |  |  | Week 4 low response, n (%): 24 (63.2) | 0.021 |  |  |
|  |  |  | Week 4 moderate-high response, n (%): 28 (40.0) |  |  |  |
|  |  | Tacrolimus | Week 2, r=–0.181 | 0.152 |  |  |
|  |  |  | Month 1, r=–0.001 | 0.993 |  |  |
|  |  |  | Month 3, r=–0.049 | 0.771 |  |  |
|  |  |  | Month 4, r=0.033 | 0.876 |  |  |
|  |  |  | Month 6, r=–0.338 | 0.010 |  |  |
| Gardiner 2021^3^ | Relationships between QFM results and immunosuppression were assessed using univariate and multivariate linear mixed effects models, with random effects fitted for patient | Tacrolimus (µg/L) | β: –5.9 (–11.3, –0.5) | 0.03 | After adjusting for other immunosuppressive medications, prednisolone dose was strongly independently associated with QFM results. There was no significant effect of other immunosuppressive agents | QFM may represent an important step towards the goal of individualizing immunosuppression dosing in lung transplant |
|  |  | Prednisolone (mg/kg) | β: –875 (–1138, –607) | <0.0001 |  |  |
|  |  | Azathioprine (mg/kg) | β: –28.7 (–79.4, 24.8) | 0.26 |  |  |
|  |  | Mycophenolate mofetil (mg/kg) | β: –1.91 (–4.69, 0.93) | 0.18 |  |  |
|  |  | Basiliximab induction | β: 0.5 (–52.1, 53.1) | 0.98 |  |  |
| Margeta 2020^4^ | Correlation matrices were used to detect association of continuous variables with QFM values | Tacrolimus (µg/L) | –0.21 | 0.08 | Tacrolimus concentration had a borderline significant negative correlation with QFM values | QFM may be useful as a tool to help guide immunosuppression dosing in kidney transplant recipients |
|  |  | Mycophenolic acid dose (mg) | –0.08 | 0.48 | No correlation between mycophenolic acid or steroid dose and QFM values |  |
|  |  | Steroid dose (mg) | 0.04 | 0.70 |  |  |
| Marx 2020^6^ | Correlations were analyzed according to Spearman | Cyclosporine A  (0–3333 ng/mL) | – | – | All 3 immunosuppressive drugs led to a strong dose-dependent inhibition in QFM values. The immune reactivity was further reduced, when cyclosporine A or tacrolimus were combined with methylprednisolone | QFM may have the potential to assess loss of global immunocompetence after transplant, but its ability to guide drug dosing on an individual basis is limited |
|  |  | Methylprednisolone (10–1000 ng/mL) | – | – |  |  |
|  |  | Tacrolimus  (0–200 ng/mL) | r=–0.23 | 0.037 |  |  |
| Mian 2018^7^ | Spearman rank order test was used to find correlations between continuous variables and expressed as correlation coefficient (ρ) | Prednisone dose | Month 1, ρ=–0.228 | 0.012 | Prednisone dose was negatively correlated with QFM values at all timepoints (*P* < 0.05). Mycophenolate doses were also inversely corelated with IFN-γ levels at Month 1 and 6. Tacrolimus levels showed some correlation but did not reach statistical significance | The QFM test may have clinical utility for defining the effect of interventions such as immunosuppression modulation based on QFM results |
|  |  |  | Month 3, ρ=–0.415 | <0.0001 |  |  |
|  |  |  | Month 6, ρ=–0.387 | 0.0005 |  |  |
|  |  | Mycophenolate sodium/ mycophenolate mofetil dose | Month 1, ρ=–0.233 | 0.025 |  |  |
|  |  |  | Month 3, ρ=–0.195 | 0.080 |  |  |
|  |  |  | Month 6, ρ=–0.282 | 0.022 |  |  |
|  |  | Tacrolimus level | Month 1, ρ=–0.176 | 0.140 |  |  |
|  |  |  | Month 3, ρ=–0.228 | 0.068 |  |  |
|  |  |  | Month 6, ρ=–0.193 | 0.170 |  |  |
| Sood 2014^11^ | Groups were compared using Dunn multiple comparison test. Linear regression analysis was used to examine any association between the assay and age, gender, and immunosuppression  levels | Tacrolimus | r^2^=0.02 | 0.35 | Tacrolimus and cyclosporin levels were not significantly associated with QFM values | The QFM test may provide an important advance in the management of patients after transplant |
|  |  | Cyclosporin | r^2^=0.16 | 0.09 |  |  |

CI, confidence interval; QFM, QuantiFERON Monitor; SOT, solid organ transplant.

**Table S6. Overall findings of the 4 identified studies that evaluated QuantiFERON^®^ Monitor in HCT recipients**

| **Study** | **Source type, study design** | **Objectives** | **Sample size** | **Timing of sampling** | **Timepoints/ timeframes assessed** | **Follow-up duration post-transplant** | **Associations with clinical outcomes** | | | **Study conclusions** |
| --- | --- | --- | --- | --- | --- | --- | --- | --- | --- | --- |
|  |  |  |  |  |  |  | **Infection** | **GvHD** | **Mortality** |  |
| Douglas 2020^12^ | Prospective observational cohort | Monitor QFM levels in alloHCT patients over the course of their transplant to profile immune recovery and correlate results with episodes of infection and GvHD | 40 | Pre- and post-transplant | Pre-conditioning, baseline, days 10, 30, 60, 90, 120, and 180 post-transplant | 180 d | Yes | No | Not reported | QFM is a promising assay to demonstrate immune recovery and predict risk of infection after alloHCT and may allow tailoring of immunosuppression, antimicrobial treatment, and prophylaxis |
| Souan 2023^13^ | Prospective observational cohort | To test whether immune-competent adult HCT recipients with CMV-specific T cells can control CMV infection or reactivation using QFM | 35 | Post-transplant | Weekly post-transplant | 100 d | Yes | Yes | Yes | QFM can predict CMV infection and mortality in HCT patients. Thus, QFM could potentially reduce transplant recipient adverse events and post-transplant costs |
| Souan 2025^14^ | Prospective observational cohort | Evaluate QFM and QF-CMV as predictors of CMV infection and GvHD in pediatric HCT recipients | 24 | Post-transplant | Weekly post-transplant | Unclear | No | No | Not reported | No statistically significant correlations between QFM values and CMV infection or GvHD. |
| Yoon 2024^15^ | Prospective observational cohort | To assess the utility of the QFM test for the prediction of early CMV infection in HCT transplant recipients | 81 | Pre-transplant, post-transplant | Pre-conditioning, 4, 8, 13, and 24 wk post-transplant | 6 mo | Yes | Not reported | Not reported | QFM can be utilized to predict the risk and burden of early CMV infection in HCT recipients 4 wk post-HCT, in conjunction with other risk factors |

Allo, allogeneic; CMV, cytomegalovirus; GvHD, graft-versus-host disease; HCT, hematopoietic cell transplant; QFM, QuantiFERON Monitor.

#### **Table S7. Clinical outcomes and results reported in the 4 identified studies that evaluated QuantiFERON^®^ Monitor in HCT recipients**

| **Study** | **Type of clinical outcome** | **QFM threshold (IU/mL)** | **HR (95% CI)*** | **OR (95% CI)^a^** | **Key results** | **Conclusions** |
| --- | --- | --- | --- | --- | --- | --- |
| Douglas 2020^12^ | Viral, bacterial, and fungal infections necessitating hospitalization admission | – | – | Day 10: 0.96 (0.93–0.99) | QFM levels varied significantly between those with active infection and those without, with higher levels in the absence of infection at a particular time point and lower levels during an infective episode (*P* < 0.001). In the sub-analysis restricted to microbiologically defined infections only, there remained a statistically significant relationship between QFM and infection at a particular timepoint *(P* < 0.001). There was a significant relationship between QFM values and incidence of infection during the neutropenic/pre-engraftment period | QFM has the potential to be a promising assay to demonstrate immune recovery and predict risk of infection post-HCT |
|  | CMV viremia (CMV DNA >1000 copies/mL) | – | – | – | There was no significant difference in QFM results between those patients with and without any detectable CMV viremia. QFM values were significantly lower in those with CMV viral load >1000 at that time point compared with those with a load <1000 or undetectable (*P* = 0.001) |  |
|  | Acute (GVHD diagnosed <100 d post-transplant) and chronic GVHD (all episodes of GVHD diagnosed thereafter) | – | – | – | There was no statistically significant difference between GVHD and QFM values (*P* = 0.21 for 3-way comparison between groups) |  |
| Souan 2023^13^ | CMV antigenemia | – | – | 0.981 (0.965–0.996) | QFM values were significantly negatively associated with CMV infection (*P* = 0.015) | QFM can predict infection and mortality, and inform implementation of pre-emptive therapies in HCT recipients post-transplant |
|  | GvHD | – | – | 1.003 (1.000–1.007) | There was a non-significant association between QFM results and GvHD incidence (*P* = 0.079) |  |
|  | Mortality | – | – | 0.991 (0.985–0.996) | QFM values were significantly negatively associated with mortality (*P* < 0.001) |  |
| Souan 2025^14^ | CMV antigenemia | - | - | 0.997 (0.991-1.003) | Non-significant negative correlation (*P* = 0.334) | No significant associations between QFM and CMV infection or GvHD |
|  | GvHD | - | - | 1.002 (1.00-1.004) | Non-significant positive correlation (*P* = 0.064) |  |
| Yoon 2024^15^ | CMV infection  (CMV DNA ≥500 IU/mL) | 86.95 | 2.15 (1.30–3.57) | – | Non-reactive QFM was associated with a higher chance of both CMV infection and high-level CMV infection at 4 wk post-HCT. In the univariate Cox regression analysis, patients with non-reactive QFM were 2.15 and 2.99 times more likely to have CMV infection and high-level CMV infection, respectively | QFM can predict risk of CMV infection as well as peak CMV viral loads in the early post-HCT period |
|  | High-level CMV infection (CMV DNA ≥5000 IU/mL) | 86.95 | 2.99 (1.65–5.44) |  |  |  |

*****For patients testing below versus above stated threshold value.

CMV, cytomegalovirus; GvHD, graft-versus-host disease; IQR, interquartile range; HCT, hematopoietic cell transplant; QFM, QuantiFERON Monitor.

# References

1. Fernandez-Ruiz M, Ruiz-Merlo T, Rodriguez-Goncer I, et al. Performance of a Global Functional Assay Based on Interferon-gamma Release to Predict Infectious Complications and Cancer After Kidney Transplantation. *Transpl Int*. 2024;37:13551. doi:10.3389/ti.2024.13551

2. NHSN. CDC/NHSN Surveillance Definitions for Specific Types of Infections. Accessed March 2025, <https://www.cdc.gov/nhsn/pdfs/pscmanual/17pscnosinfdef_current.pdf>

3. Gardiner BJ, Lee SJ, Cristiano Y, et al. Evaluation of Quantiferon(R)-Monitor as a biomarker of immunosuppression and predictor of infection in lung transplant recipients. *Transplant infectious disease : an official journal of the Transplantation Society*. Jun 2021;23(3):e13550. doi:10.1111/tid.13550

4. Margeta I, Mareković I, Pešut A, et al. Evaluation of cell-mediated immune response by QuantiFERON Monitor Assay in kidney transplant recipients presenting with infective complications. *Medicine*. Jul 2 2020;99(27):e21010. doi:10.1097/md.0000000000021010

5. Humar A, Michaels M. American Society of Transplantation recommendations for screening, monitoring and reporting of infectious complications in immunosuppression trials in recipients of organ transplantation. *Am J Transplant*. Feb 2006;6(2):262–274. doi:10.1111/j.1600-6143.2005.01207.x

6. Marx S, Adam C, Mihm J, Weyrich M, Sester U, Sester M. A Polyclonal Immune Function Assay Allows Dose-Dependent Characterization of Immunosuppressive Drug Effects but Has Limited Clinical Utility for Predicting Infection on an Individual Basis. *Frontiers in immunology*. 2020;11:916. doi:10.3389/fimmu.2020.00916

7. Mian M, Natori Y, Ferreira V, et al. Evaluation of a novel global immunity assay to predict infection in organ transplant recipients. *Clinical infectious diseases : an official publication of the Infectious Diseases Society of America*. Apr 17 2018;66(9):1392–1397. doi:10.1093/cid/cix1008

8. Solidoro P, Curtoni A, Patrucco F, et al. QuantiFERON(®) Monitor Test as a Potential Tool for Stratifying Patients by Infection Risk and Tailoring Follow-Up Care in Lung Transplant Recipients: A Single-Center Retrospective Experience. *Microorganisms*. Feb 1 2025;13(2)doi:10.3390/microorganisms13020316

9. Sood S, Yu L, Visvanathan K, Angus PW, Gow PJ, Testro AG. Immune function biomarker QuantiFERON-monitor is associated with infection risk in cirrhotic patients. *World journal of hepatology*. Dec 18 2016;8(35):1569–1575. doi:10.4254/wjh.v8.i35.1569

10. Sood S, Haifer C, Yu L, et al. A novel immune function biomarker identifies patients at risk of clinical events early following liver transplantation. *Liver transplantation : official publication of the American Association for the Study of Liver Diseases and the International Liver Transplantation Society*. Apr 2017;23(4):487–497. doi:10.1002/lt.24730

11. Sood S, Cundall D, Yu L, et al. A novel biomarker of immune function and initial experience in a transplant population. *Transplantation*. Apr 27 2014;97(8):e50–1. doi:10.1097/tp.0000000000000078

12. Douglas AP, Yu L, Sundararajan V, et al. The QuantiFERON Monitor(®) assay is predictive of infection post allogeneic hematopoietic cell transplantation. *Transplant infectious disease : an official journal of the Transplantation Society*. Jun 2020;22(3):e13260. doi:10.1111/tid.13260

13. Souan L, Jazar HA, Nashwan S, Sughayer MA. QuantiFERON-CMV and monitor predict cytomegalovirus, mortality, and graft-versus-host disease in transplant recipients. *Journal of medical virology*. Nov 2023;95(11):e29250. doi:10.1002/jmv.29250

14. Souan L, Rihani R, Sughayer MA. Predicting cytomegalovirus infection and graft-versus-host disease using QuantiFERON-CMV and Monitor in pediatric transplants: a proof-of-concept study. *Therapeutic advances in hematology*. 2025;16:20406207251316680. doi:10.1177/20406207251316680

15. Yoon E, Shin S, Choi S, et al. QuantiFERON monitor predicts early cytomegalovirus infection and viral burden in allogeneic hematopoietic stem cell transplantation. *Transplant infectious disease : an official journal of the Transplantation Society*. Jul 9 2024:e14328. doi:10.1111/tid.14328
